# Testing dynamic correlations and nonlinearity in bivariate time series through information measures and surrogate data analysis

**DOI:** 10.3389/fnetp.2024.1385421

**Published:** 2024-05-21

**Authors:** Helder Pinto, Ivan Lazic, Yuri Antonacci, Riccardo Pernice, Danlei Gu, Chiara Barà, Luca Faes, Ana Paula Rocha

**Affiliations:** ^1^ Departamento de Matemática, Faculdade de Ciências, Universidade do Porto, Porto, Portugal; ^2^ Centro de Matemática da Universidade do Porto (CMUP), Porto, Portugal; ^3^ Faculty of Technical Sciences, University of Novi Sad, Novi Sad, Serbia; ^4^ Department of Engineering, University of Palermo, Palermo, Italy; ^5^ Beijing Jiaotong University, Beijing, China

**Keywords:** complex systems, coupling, information storage, information theory, mutual information rate, surrogate analysis

## Abstract

The increasing availability of time series data depicting the evolution of physical system properties has prompted the development of methods focused on extracting insights into the system behavior over time, discerning whether it stems from deterministic or stochastic dynamical systems. Surrogate data testing plays a crucial role in this process by facilitating robust statistical assessments. This ensures that the observed results are not mere occurrences by chance, but genuinely reflect the inherent characteristics of the underlying system. The initial process involves formulating a null hypothesis, which is tested using surrogate data in cases where assumptions about the underlying distributions are absent. A discriminating statistic is then computed for both the original data and each surrogate data set. Significantly deviating values between the original data and the surrogate data ensemble lead to the rejection of the null hypothesis. In this work, we present various surrogate methods designed to assess specific statistical properties in random processes. Specifically, we introduce methods for evaluating the presence of autodependencies and nonlinear dynamics within individual processes, using Information Storage as a discriminating statistic. Additionally, methods are introduced for detecting coupling and nonlinearities in bivariate processes, employing the Mutual Information Rate for this purpose. The surrogate methods introduced are first tested through simulations involving univariate and bivariate processes exhibiting both linear and nonlinear dynamics. Then, they are applied to physiological time series of Heart Period (RR intervals) and respiratory flow (RESP) variability measured during spontaneous and paced breathing. Simulations demonstrated that the proposed methods effectively identify essential dynamical features of stochastic systems. The real data application showed that paced breathing, at low breathing rate, increases the predictability of the individual dynamics of RR and RESP and dampens nonlinearity in their coupled dynamics.

## 1 Introduction

The increasing accessibility of detailed and extensive signal recordings from various sources is opening the way to the representation of complex systems through network structures. For example, in physiology, the traditional reductionist approach, which involves studying the function of an organ system in isolation, is now complemented by a holistic investigation of collective interactions among diverse organ systems in the field of Network Physiology (Bashan et al., 2012; Ivanov et al., 2017; Ivanov, 2021). Network Physiology falls within the broader domain of Network Science, an expansive interdisciplinary field dedicated to advancing theoretical and practical methodologies to improve the comprehension of natural and artificial networks characterized by hierarchical structures (Barabási, 2013).

Within the Network Physiology approach, the human body is commonly represented as a graph, encoding the observed dynamical system with distinct nodes (e.g., organ systems) connected by edges that map functional dependencies (Lehnertz et al., 2020). The primary methods employed to analyze these dependencies include state-space interdependence (Arnhold et al., 1999; Faes et al., 2008a), correlation analyses (Li et al., 2009), and the application of Granger causality (GC) in both the time and frequency domains (Bressler and Seth, 2011). Information dynamics (Lizier, 2012) is a versatile framework that includes most of these approaches and has been widely used to characterize the interdependence of coupled systems in various fields, especially in physiology (Widjaja et al., 2015; Javorka et al., 2018). Entropy-based measures have been demonstrated to be effective in evaluating physiological interactions in cardiorespiratory (Widjaja et al., 2015), cardiovascular (Faes et al., 2019a), and cerebrovascular systems (Bari et al., 2023). These measures assess complexity (Pincus, 1991; Faes et al., 2017) and information stored within a process (Faes et al., 2019b), as well as directed information transfer between processes (Porta and Faes, 2013). Another key characteristic to assess in physiological interactions is the dynamic coupling between two processes which can be evaluated through the computation of the Mutual Information Rate (MIR) (Barà et al., 2023; Pinto et al., 2023). This information-theoretic measure quantifies the degree of information shared between two systems, providing a measure of their dynamic coupling strength and revealing the interdependencies within their dynamic behavior.

Nevertheless, the inherent variability in dynamic systems, particularly physiological ones, requires proper statistical tests to evaluate the significance of estimated information measures. Without such testing, the reliability of these measures remains uncertain. Typically, this assessment involves the use of surrogate analysis methods (Lucio et al., 2012; Lancaster et al., 2018). Surrogate data testing provides a versatile framework applicable to signals that arise from any physical system, allowing the exploration of fundamental questions about the system. Moreover, incorporating information dynamic measures, such as IS and MIR, into these methods enables the testing of specific hypotheses related to the observed data (Jamšek et al., 2010; Cliff et al., 2021). In typical surrogate analysis methods, a null hypothesis is formulated, assuming the absence of a specific characteristic to be tested in the observed data. Subsequently, surrogate data consistent with the null hypothesis is generated, and a discriminating statistic is computed for both the original and surrogate data. If the discriminating statistic of the original data significantly deviates from the surrogate distribution, the null hypothesis is rejected (Schreiber and Schmitz, 2000; Lancaster et al., 2018).

In time series analysis, several methods have emerged to assess the temporal statistical structure and the interaction between interconnected dynamic processes (Theiler et al., 1992; Palus, 1996; Popivanov and Mineva, 1999; Faes et al., 2008b; Lancaster et al., 2018; Calderón-Juárez et al., 2023). In this study, surrogate-based tests were implemented, using IS as a discriminating statistic, along with shuffling procedures and the Iterative Amplitude Adjusted Fourier Transform (IAAFT) algorithm (Schreiber and Schmitz, 1996) to assess the presence of self-dependencies and nonlinear dynamics in univariate processes. Additionally, these tests were extended to bivariate systems, incorporating MIR as a discriminating statistic, to investigate coupling and nonlinearity in coupled systems. All the proposed methods were validated through simulations involving both univariate processes and bivariate systems with linear and nonlinear dynamics. Finally, the framework is applied to a simple exemplary application in cardiovascular and cardiorespiratory time series, collected from healthy subjects monitored during different breathing conditions, to assess changes in the strength of the cardiorespiratory coupling and the extent of nonlinearities.

## 2 Framework for the surrogate data analysis of dynamic correlations and nonlinearity

Within the surrogate family, there are two distinct approaches: typical realizations and constrained realizations (Lancaster et al., 2018). In typical realizations, a model is fitted to the data, such as estimating the coefficients of an autoregressive (AR) model. Subsequently, Monte Carlo realizations of this model are generated for comparison with the data. However, a drawback of this method is that the user needs to have prior knowledge of the model and its parameters. On the other hand, constrained realizations are computed directly from the data, producing surrogates that replicate all characteristics of the original data except for the specific property being tested (Theiler and Prichard, 1996). Although the constrained realization approach, commonly known as surrogate data, is more widely adopted, it is essential to acknowledge that this method is specifically tailored for hypothesis testing. In contrast to typical realizations, it cannot be employed for the estimation of confidence intervals (Theiler and Prichard, 1996).

In this constrained realization approach, two fundamental steps need to be addressed: the selection of a suitable discriminating statistic and the proper formulation of the null hypothesis to be tested. This ensures that when rejection occurs, it is possible to conclude that the null hypothesis is not suitable to describe the data. Conversely, not rejecting the null hypothesis does not inherently mean its acceptance. Surrogates exclusively test the null hypothesis specified for them, without confirming its accuracy. Since surrogates do not always have a Gaussian distribution, it is more robust to use a non-parametric test where the significance level is determined, for example, by the 95th percentile of the surrogate distribution. The required number of surrogates depends on whether a one-sided or two-sided test is being conducted. In a one-sided test, the null hypothesis is rejected only if the distribution of surrogate values of the discriminating statistic deviates from those calculated in the original data in one specified direction—either lower or higher. On the contrary, a two-sided test allows for testing the possibility of the discriminating statistic being either higher or lower in the surrogates than in the original data (Theiler et al., 1992; Schreiber and Schmitz, 2000).

The following sections provide a detailed overview of surrogate methods for testing the presence of specific statistical structures in both univariate and bivariate processes. Firstly, in Section 2.1, the discriminating statistics employed in this work, Information Storage (IS) and Mutual Information Rate (MIR), are described in detail, along with the nearest neighbor estimation of these information measures. Then, in Sections 2.2 and 2.3, respectively, methods for assessing the presence of self-dependencies and nonlinearities in univariate processes are introduced. Lastly, Sections 2.4 and 2.5 discuss, respectively, surrogates for testing the existence of dynamic coupling and the nonlinear dynamics in bivariate systems.

### 2.1 Discriminating statistics

#### 2.1.1 Information-theoretic preliminaries

As information-theoretic analysis of random processes relies on information measures applied to random variables, we will begin by reviewing the entropy measures adopted for describing general static random variables. Subsequently, these measures are extended to stochastic processes representing networks of interacting dynamical systems, enabling the formulation of Information Storage (IS) and Mutual Information Rate (MIR), which are later used as discriminating statistics.

One of the most well-known and widely used information measures is entropy (Shannon, 1948; Cover, 1999). Entropy serves as a measure of uncertainty or disorder associated with a random variable. For a discrete random variable *X* with a probability mass function *p*(*x*), the entropy *H*(*X*) is defined by

$$H\left(X\right)=E\left[\ln\frac{1}{p\left(x\right)}\right],$$ (1)

where E[.] represents the expectation operator. Since it is formulated in terms of the natural logarithm, the entropy is measured in natural units (*nats*). High entropy values indicate high uncertainty, while low entropy suggests more predictability.

Moving beyond single variables, conditional entropy (CE), denoted as *H*(*Y*|*X*), measures the remaining uncertainty of a random variable *Y* given the knowledge of *X* and is defined as

$$H\left(Y|X\right)=-E\left[\ln\left(\frac{p\left(x,y\right)}{p\left(x\right)}\right)\right],$$ (2)

where *p* (*x*, *y*) is the joint probability mass function of *X* and *Y*.

The amount of information shared between two random variables can be measured by Mutual Information (MI), which is crucial for understanding the relationships and dependencies between variables (Cover, 1999). MI, denoted as *I*(*X*;*Y*), between *X* and *Y* is defined as the expected value of the pointwise mutual information

$$I\left(X;Y\right)=E\left[\ln\left(\frac{p\left(x,y\right)}{p\left(x\right)p\left(y\right)}\right)\right].$$ (3)

The logarithm term inside the expectation measures the ratio of the joint probability to the product of the marginals, reflecting the departure from independence. Positive values of mutual information indicate dependence, while zero suggests independence between *X* and *Y*.

#### 2.1.2 Information measures in dynamic processes

This Section describes the use of the information measures defined in Section 2.1.1, applied by taking as arguments proper combinations of the present and past states of the stochastic processes representative of a network of interacting dynamical systems, to formulate a framework quantifying auto-dependencies in univariate processes through IS and dynamic coupling in bivariate systems via the MIR.

Consider two stationary, ergodic, potentially interacting stochastic processes, *X* and *Y*. Let us assume that the realization of system states is suitably described as a multivariate stationary stochastic process *S* = [*X*, *Y*]. In a temporal reference frame where *n* denotes the present time, *Y*_*n*_ represents the current state of *Y*, and $Y_{n}^{-}=\left[Y_{n-1},Y_{n-2},\ldots \right]$ describes its past. The same notation applies to the process *X*. This simple operation of separating the present from the past allows us to consider the flow of time and study causal interactions within and between processes by examining the statistical dependencies among these variables (Faes and Porta, 2014). Furthermore, by adopting the Markov assumption, which posits a finite-length memory for the investigated system, the past of each process is approximated by vector variables with dimension 1 × *q*, i.e., $X_{n}^{q}\approx \left[X_{n-1},X_{n-2},\ldots ,X_{n-q}\right]$ and $Y_{n}^{q}\approx \left[Y_{n-1},Y_{n-2},\ldots ,Y_{n-q}\right]$ .

##### 2.1.2.1 Information storage

IS can be interpreted as a measure of the process regularity and for the process X can be defined as (Faes et al., 2019b)

$$S_{X}=I\left(X_{n};X_{n}^{q}\right)=E\left[\ln\frac{p\left(x_{n},x_{n-1},\ldots ,x_{n-q}\right)}{p\left(x_{n}\right)p\left(x_{n-1},x_{n-2},\ldots ,x_{n-q}\right)}\right].$$ (4)

Here, the expectation is calculated across various instances $\left(x_{n},x_{n-1},\ldots ,x_{n-q}\right)$ of the random variables $\left(X_{n},X_{n-1},\ldots X_{n-q}\right)$ . Considering the dynamic evolution of a time-evolving system, the concept of IS complements a widely recognized measure of system complexity, which is quantified as the entropy rate. This rate, for ergodic processes, is defined as the conditional entropy of the current state given its past states, denoted as $H_{X}=H\left(X_{n}|X_{n}^{q}\right)$ (Cover, 1999; Martins et al., 2020). Thus, IS can be rewritten as

$$S_{X}=H\left(X_{n}\right)-H_{X}.$$ (5)

Moreover, given that $H\left(X_{n}|X_{n}^{q}\right)=H\left(X_{n},X_{n}^{q}\right)-H\left(X_{n}^{q}\right)$ , the IS can be reformulated exclusively in terms of entropy, i.e.,

$$S_{X}=H\left(X_{n}\right)+H\left(X_{n}^{q}\right)-H\left(X_{n},X_{n}^{q}\right).$$ (6)

It is worth noting that the IS for the *Y* process is defined in exactly the same way, simply by replacing *X* with *Y* in the previous expressions. As the entropy measures previously defined, the IS is also expressed in *nats*.

##### 2.1.2.2 Mutual information rate

The MIR of the bivariate process **S** = {*X*, *Y*} is defined as the limit, if it exists, of the rate at which the MI between dynamic sequences taken from *X* and *Y* increases over time (Komaee, 2020; Miao et al., 2020)

$$I_{X;Y}=\lim_{q\to \infty }\frac{1}{q}I\left(X_{n}^{q};Y_{n}^{q}\right).$$ (7)

The MIR serves as a dynamic measure for the information shared per unit of time between two dynamical systems and was adopted in various forms to quantify dynamic interactions among physiological processes (Baptista and Kurths, 2008; Mijatovic et al., 2021; Faes et al., 2022). This measure of dynamic coupling can be decomposed in terms of other information-theoretic measures, offering valuable insights into the dynamics of each process and the coupling relationships within the bivariate system (Barà et al., 2023). A possible decomposition of MIR is

$$I_{X;Y}=H_{X}+H_{Y}-H_{X,Y},$$ (8)

where *H*_*X*_ and *H*_*Y*_ denote the entropy rate of *X* and *Y*, respectively, and *H*_*X*,*Y*_ their joint entropy rate. Exploiting the fact that the conditional entropy terms can be written as the difference between two entropies and substituting the above-defined terms in Eq. 8, the MIR can be reformulated as follows

$$I_{X;Y}=H\left(X_{n},X_{n}^{q}\right)-H\left(X_{n}^{q}\right)+H\left(Y_{n},Y_{n}^{q}\right)-H\left(Y_{n}^{q}\right)-H\left(X_{n},Y_{n},X_{n}^{q},Y_{n}^{q}\right)+H\left(X_{n}^{q},Y_{n}^{q}\right),$$ (9)

favoring the estimation of the MIR as the sum of entropy terms, measured also in *nats*.

#### 2.1.3 Estimation of information measures

In this work, the most widely used non-parametric estimator for continuous random variables was applied, the *k*-nearest neighbor (KNN). Due to its ability to adjust resolution dynamically by altering the distance scale based on the underlying probability distribution (Victor, 2002), and its potential for bias compensation through distance projection (Kraskov et al., 2004), the nearest-neighbor technique has become increasingly popular in recent years for estimating entropy measures in the analysis of time series.

##### 2.1.3.1 Nearest-neighbor estimation

The KNN estimation approach derives an approximation of the probability distribution by analyzing the statistical properties of the distances among neighboring points within the multidimensional spaces defined by the observed variables. This approach is grounded in the results presented in (Kozachenko and Leonenko, 1987), which assert that the mean Shannon information content of a general *d*-dimensional random variable *V* can be estimated from the set of *N* observations {*v*_1_, *v*_2_, … , *v*_*N*_} of the variable as

$$-E\left[\ln p\left(v_{n}\right)\right]=\psi \left(N\right)-\psi \left(k\right)+d\left\langle \ln\varepsilon_{n}\right\rangle ,$$ (10)

where *ψ* denotes the digamma function, *ɛ*_*n*_ represents twice the distance between *v*_*n*_ and its *k*th nearest neighbor, determined using the maximum norm and ⟨.⟩ denotes the average over all the *N* realizations of *V*. It is straightforward, from Eq. 10, to obtain the formula for the KNN estimate of the entropy for *X*_*n*_, calculated based on the time series {*x*_1_, *x*_2_, … , *x*_*N*_}

$$H\left(X_{n}\right)=\psi \left(N\right)-\psi \left(k\right)+\left\langle \ln\varepsilon_{n}\right\rangle .$$ (11)

According to Eqs 6, 9, the IS and MIR can be computed as a combination of entropy terms. Nevertheless, these entropy terms are calculated in spaces with distinct dimensions, and applying the same neighbor search procedure uniformly across all spaces would yield distinct distance lengths when approximating the probability density in different dimensions. This divergence in distance lengths could introduce estimation biases that cannot be rectified by taking the entropy differences. Hence, in order to keep the same distance length in all explored spaces, the approach discussed in (Kraskov et al., 2004) was used. This method conducts a neighbor search only in the highest-dimensional space and then projects the distances identified in this space to the lower-dimensional spaces. This ensures that these distances serve as the range for counting neighbors in each respective space. Specifically, for the IS estimation, the KNN estimate of $H\left(X_{n},X_{n}^{q}\right)$ is computed through the neighbor search

$$H\left(X_{n},X_{n}^{q}\right)=\psi \left(N\right)-\psi \left(k\right)+\left(q+1\right)\left\langle \ln\varepsilon_{n}\right\rangle ,$$ (12)

with *ɛ*_*n*_ representing twice the distance from $\left[x_{n},x_{n}^{q}\right]$ to its *k*th nearest neighbor. Subsequently, based on the calculated distances *ɛ*_*n*_, the entropies in the lower-dimensional spaces are assessed using a range search.

$$H\left(X_{n}^{q}\right)=\psi \left(N\right)-\left\langle \psi \left(N_{X_{n}^{q}}+1\right)\right\rangle +q\left\langle \ln\varepsilon_{n}\right\rangle ,$$ (13a)

$$H\left(X_{n}\right)=\psi \left(N\right)-\left\langle \psi \left(N_{X_{n}}+1\right)\right\rangle +\left\langle \ln\varepsilon_{n}\right\rangle ,$$ (13b)

where $N_{X_{n}}$ and $N_{X_{n}^{q}}$ are the number of points whose distance from *x*_*n*_ and $x_{n}^{q}$ , respectively, is smaller than *ɛ*_*n*_/2. Therefore, IS is obtained subtracting Eq. 12 from the sum of Eq. 13

$$S_{X}=\psi \left(N\right)+\psi \left(k\right)-\left\langle \psi \left(N_{X_{n}^{q}}+1\right)\right\rangle -\left\langle \psi \left(N_{X_{n}}+1\right)\right\rangle .$$ (14)

Analogously, for the MIR estimation, considering the bivariate system **S** = {*X*, *Y*}, the KNN estimate of the entropy in the higher space, i.e., $\left[X_{n},Y_{n},X_{n}^{q},Y_{n}^{q}\right]$ , is computed through the neighbor search as follows

$$H\left(X_{n},Y_{n},X_{n}^{q},Y_{n}^{q}\right)=-\psi \left(k\right)+\psi \left(N\right)+2\left(q+1\right)\left\langle \log\varepsilon_{n}\right\rangle$$ (15)

where, in this case, *ɛ*_*n*_ denotes twice the distance from $\left[x_{n},y_{n},x_{n}^{q},y_{n}^{q}\right]$ to its *k*th nearest neighbor. Following this, the entropies in the lower-dimensional spaces are determined through a range search using *ɛ*_*n*_. Particularly, in this framework, $H\left(X_{n},X_{n}^{q}\right)$ and $H\left(X_{n}^{q}\right)$ are computed respectively as.

$$H\left(X_{n},X_{n}^{q}\right)=\psi \left(N\right)-\left\langle \psi \left(N_{X_{n}X_{n}^{q}}+1\right)\right\rangle +\left(q+1\right)\left\langle \ln\varepsilon_{n}\right\rangle ,$$ (16a)

$$H\left(X_{n}^{q}\right)=\psi \left(N\right)-\left\langle \psi \left(N_{X_{n}^{q}}+1\right)\right\rangle +q\left\langle \ln\varepsilon_{n}\right\rangle ,$$ (16b)

where $N_{X_{n}X_{n}^{q}}$ and $N_{X_{n}^{q}}$ are the number of points whose distance from $\left[x_{n},x_{n}^{q}\right]$ and $x_{n}^{q}$ is smaller than *ɛ*_*n*_/2, respectively. Similarly, $H\left(Y_{n},Y_{n}^{q}\right)$ and $H\left(Y_{n}^{q}\right)$ can be derived by replacing *X* with *Y* in the corresponding above equations, thus obtaining.

$$H\left(Y_{n},Y_{n}^{q}\right)=\psi \left(N\right)-\left\langle \psi \left(N_{Y_{n}Y_{n}^{q}}+1\right)\right\rangle +\left(q+1\right)\left\langle \ln\varepsilon_{n}\right\rangle ,$$ (17a)

$$H\left(Y_{n}^{q}\right)=\psi \left(N\right)-\left\langle \psi \left(N_{Y_{n}^{q}}+1\right)\right\rangle +q\left\langle \ln\varepsilon_{n}\right\rangle ,$$ (17b)

$N_{Y_{n}Y_{n}^{q}}$ and $N_{Y_{n}^{q}}$ are the number of points whose distance from $\left[y_{n},y_{n}^{q}\right]$ and $y_{n}^{q}$ is smaller than *ɛ*_*n*_/2. Then, the joint entropy of the past state of both processes can be computed as:

$$H\left(X_{n}^{q},Y_{n}^{q}\right)=\psi \left(N\right)-\left\langle \psi \left(N_{X_{n}^{q}Y_{n}^{q}}+1\right)\right\rangle +2q\langle \log\left(\varepsilon_{n}\right)\rangle ,$$ (18)

where $N_{X_{n}^{q},Y_{n}^{q}}$ denote the number of points whose distance from $\left[x_{n}^{q},y_{n}^{q}\right]$ is smaller than *ɛ*_*n*_/2. Finally, the KNN estimate of MIR is derived plugging Eqs 15–18 in Eq. 9, thus obtaining

$$I_{X;Y}=\psi \left(k\right)+\left\langle \psi \left(N_{X_{n}^{q}}+1\right)+\psi \left(N_{Y_{n}^{q}}+1\right)-\psi \left(N_{X_{n}X_{n}^{q}}+1\right)-\psi \left(N_{Y_{n}Y_{n}^{q}}+1\right)-\psi \left(N_{X_{n}^{q}Y_{n}^{q}}+1\right)\right\rangle .$$ (19)

### 2.2 Surrogates for auto-dependencies in univariate processes

Many physical and biological systems exhibit complex dynamic behaviors resulting from the presence of self-sustained oscillators, interacting subsystems, and feedback loops responding to both internal and external stimuli (Pincus, 1991; Donges et al., 2009; Chialvo, 2010). Therefore, assessing the existence of self-dependencies in these types of systems is crucial. In this context, for detecting auto-dependencies, in this work a surrogate method that employs the IS as a discriminating statistic is proposed. Let us consider the time series **x** = {*x*_1_, *x*_2_, *x*_3_, … , *x*_*N*_} as a realization of length *N* of the univariate stochastic process *X*. Moreover, assuming an embedding dimension of *q*, the estimation of IS, as described in Section 2.1.2.1, relies on the (*N* − *q*) × (*q* + 1) observation matrix, defined as

$$\left[\begin{array}{ccccc} x_{q+1} & x_{q} & x_{q-1} & \ldots & x_{1}\\ x_{q+2} & x_{q+1} & x_{q} & \ldots & x_{2}\\ \vdots & \vdots & \vdots & \vdots & \vdots \\ x_{N} & x_{N-1} & x_{N-2} & \ldots & x_{N-q} \end{array}\right].$$ (20)

This matrix plays a central role in the proposed surrogate method.

*Null Hypothesis.* The time series **x** comes from a process that does not exhibit self-dependencies; equivalently, *S*_*X*_ = 0.

**FIGURE 1:**
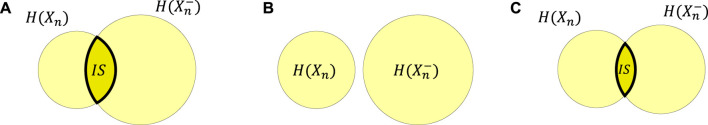
Schematic description of surrogate frameworks proposed for assessing the presence of self-dependencies and nonlinearities in univariate random processes: **(A)** presents the Venn diagram of the dependency between the past and the present of the original time series, measured by the IS. In **(B)**, the proposed surrogate procedure to assess the presence of self-dependencies will generate time series where the dependencies between the present and the past are destroyed. In this case, the Venn diagram is presented as two non-intersecting circles. In **(C)**, the surrogate method for detecting nonlinearities will create time series where the nonlinear correlation between the past and the present state of the process is destroyed. Under these conditions, the dependency between the past and the present is not totally destroyed but is equivalent to that arising from a time series with linear correlations only, so the Venn diagram presents a reduced intersection.

*Algorithm.* Perform a random shuffle on the column corresponding to the present state of the time series **x**, which is the first column of the observation matrix ${\left[x_{q+1},x_{q+2},\ldots x_{N}\right]}^{\text{T}}$ (T represents matrix transpose), thereby eliminating any dependency between the present samples and their past (see Figure 1B). After this shuffling process, the IS is estimated. This procedure is repeated *M* times to obtain the distribution of IS for the surrogates. Finally, a percentile-based test is performed, where the null hypothesis is rejected if, with a significance level *α*, the IS estimated for the original process exceeds the (1 − *α*)^th^ percentile of the surrogate distribution, indicating that the process does have self-dependencies.

### 2.3 Surrogates for nonlinear dynamics in univariate processes

The presence of nonlinear dynamics was assessed using the Iterative Amplitude Adjusted Fourier Transform (IAAFT) surrogates introduced by (Schreiber and Schmitz, 1996). This method involves the iterative replacement of Fourier amplitudes with the correct values and rescaling the distribution to achieve a closer match between the distribution and the power spectrum in the original data and the surrogates (Lancaster et al., 2018).

*Null Hypothesis.* The time series **x** comes from a process that exclusively exhibit linear dynamics.

*Algorithm*. The algorithm involves an iterative process:

1. Store a sorted list of the values of the time series **x** and the squared amplitudes of its Fourier transform $S_{k}^{2}={\left|\sum_{n=0}^{N-1}x_{n}e^{i2\pi kn/N}\right|}^{2}$ , with *k* = 1, … , *N*.
2. Randomly shuffle (without replacement) **x** ^(0)^, where the superscript ^(0)^ denotes the iteration number.
3. **x** ^(*i*)^ is adjusted to match the desired sample power spectrum. This involves performing a Fourier transform on **x** ^(*i*)^, replacing the squared amplitudes $S_{k}^{2,\left(i\right)}$ with $S_{k}^{2}$ , and subsequently applying the inverse Fourier Transform. The phases of the complex Fourier components are retained. Therefore, this step ensures the correct spectrum, but it typically results in a modified distribution.
4. Arrange the derived series in a ranked order to precisely match the values of **x**. Unfortunately, this results in a further modification of the spectrum in **x** ^(*i*+1)^.
5. Repeat steps 3 and 4 to ensure that the power spectrum and distribution are as close to the original data as possible. In this work, seven iterations were used to avoid high computational costs, and indeed, this number of iterations is sufficient to observe a low percentage of false rejections (Schreiber and Schmitz, 1996).

This iterative procedure is repeated *M* times to obtain the distribution of the IS of the 100 surrogates. The *null hypothesis* is rejected, with a significance level of *α*, when the IS estimated in the original process exceeds the (1 − *α*)^th^ percentile of the surrogate distribution, indicating that the data come from a nonlinear process. Figure 1C represents graphically this surrogate procedure in terms of a Venn diagram. The IAFFT algorithm generates a time series in which the nonlinear correlation between the past and present state of the process is disrupted. In this case, the dependency between the past and the present is not entirely destroyed but is reduced compared to the original case presented in Figure 1A, resulting in a reduced intersection in the Venn diagram.

### 2.4 Surrogates for the presence of dynamic coupling in bivariate processes

Consider that **x** = {*x*_1_, *x*_2_, … , *x*_*N*_} and **y** = {*y*_1_, *y*_2_, … , *y*_*N*_} are realizations of length *N* for the stochastic processes *X* and *Y*, respectively. Similar to the approach with IS, described in Section 2.2, assuming an embedding dimension of *q*, the estimation of the MIR relies on an (*N* − *q*) × 2 (*q* + 1) observation matrix given by

$$\left[\begin{array}{cccccccc} x_{q+1} & x_{q} & \ldots & x_{1} & y_{q+1} & y_{q} & \ldots & y_{1}\\ x_{q+2} & x_{q+1} & \ldots & x_{2} & y_{q+2} & y_{q+1} & \ldots & y_{2}\\ \vdots & \vdots & \vdots & \vdots & \vdots & \vdots & \vdots & \vdots \\ x_{N} & x_{N-1} & \ldots & x_{N-q} & y_{N} & y_{N-1} & \ldots & y_{N-q} \end{array}\right],$$ (21)

which is exploited for generating surrogate time series.

*Null Hypothesis*. The time series **x** and **y** are realizations of independent processes, or, equivalently, I_*X*;*Y*_ = 0.

**FIGURE 2:**
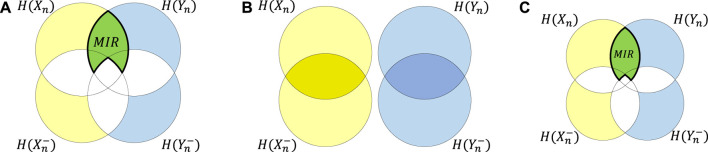
Schematic description of surrogate frameworks proposed for assessing the presence of coupling, nonlinearities, and temporal correlations in bivariate random processes: **(A)** presents the Venn diagram of the coupling between two generic processes *X* (yellow) and *Y* (blue) measured by MIR, represented in green. In **(B)**, the proposed surrogate procedure to assess the presence of coupling will generate time series where the dependencies between the two processes are destroyed, but dependencies between the past and the present of each process are maintained. In **(C)**, the surrogate method for detecting nonlinearities will create a time series where the nonlinear correlation between the past and the present state of the process is destroyed. For this scenario, the MIR between the processes is not completely lost but is inferior to the original case **(A)**, so the Venn diagram presents a reduced MIR intersection.

*Algorithm*. Shuffle the rows of the (*N* − *q*) × (*q* + 1) sub-matrix of the observation matrix defined as

$$\left[\begin{array}{cccc} x_{q+1} & x_{q} & \ldots & x_{1}\\ x_{q+2} & x_{q+1} & \ldots & x_{2}\\ \vdots & \vdots & \vdots & \vdots \\ x_{N} & x_{N-1} & \ldots & x_{N-q} \end{array}\right],$$ (22)

thus destroying the coupling while maintaining the internal dynamics of *X* and *Y*, as illustrated in Figure 2B. It is important to note that the matrix shown relates to process *X* for illustrative purposes only; likewise, choosing the sub-matrix for process *Y* would yield the same results. After this step, the shuffled observation matrix is used to estimate the MIR. This procedure is iterated *M* times to derive the surrogate distribution of the MIR, and the *null hypothesis* is rejected, with a significance level *α*, if the MIR value estimated in the original time series exceeds the (1 − *α*)^th^ percentile of the surrogate distribution, indicating that the processes are coupled.

### 2.5 Surrogates for the assessment of nonlinear interactions in bivariate processes

Fourier Transform (FT) surrogates, along with any other surrogate types wherein the phases are randomized, such as IAFFT introduced in Section 2.3, can be employed to investigate nonlinear dependencies in multivariate data. Let consider **x** = {*x*_1_, *x*_2_, … , *x*_*N*_} and **y** = {*y*_1_, *y*_2_, … , *y*_*N*_} as realizations of length *N* from the bivariate stochastic system *S* = {*X*, *Y*}.

*Null Hypothesis*. The time series **x** and **y** are realizations of a bivariate process exhibiting exclusively linear correlations, or equivalently, the data constitutes a realization of a multivariate Gaussian process.

*Algorithm*. The algorithm is similar to the previously presented IAAFT in Section 2.3. The only difference in the procedure lies in step 2, where, instead of applying a random permutation to both series, the same random number in the range of [0, 2*π*] is added to the phases of both processes (Lancaster et al., 2018). This approach preserves not only the power spectrum but also the cross-spectrum between signals, as highlighted by Prichard and Theiler (1994). In this case, the MIR between the processes is not completely lost but is inferior to the original, illustrated in Figure 2A, so the Venn diagram presents a reduced MIR intersection on Figure 2C.

As before, this procedure is repeated *M* times to obtain the distribution of the MIR of the *M* surrogates. The *null hypothesis* is rejected, with a significance level *α*, when the MIR estimated in the original process exceeds the 95th percentile of the surrogate distribution, indicating the presence of nonlinear dependencies between the processes constituting the bivariate system.

## 3 Toolbox description

In this section, we present the Surrogates for Information Dynamics measures toolbox (SID) with the utilized measures and surrogate techniques previously outlined. The toolbox is designed within MATLAB and is equipped with all essential functions required for the described investigation within this work, with some demo scripts in order to demonstrate its functionality.

The used functions are within the */SID/functions* folder, which can be grouped into four distinct categories:

• data manipulation,
• KNN supporting functions,
• measure estimation,
• surrogate generation algorithms.

Details for each individual provided function are covered in Table 1. Here, the supporting functions for efficient KNN algorithm computations are obtained from (Lindner et al., 2011) and are given as compiled MEX files for Windows and Linux systems.

**TABLE 1:** SID toolbox function contents.

Function name	Description
data manipulation	
surr_SetLag	Creates the desired embedding vector
surr_ObsMat	Forms the observation matrix
KNN supporting functions	
nn_prepare	Construct a tree structure for nearest neighbor search
nn_search	Apply nearest neighbor search for a given query
range_search	Find points that are within a range for a given query
measure estimation	
surr_ISknn	K-nearest neighbor estimation of Information Storage
surr_MIRknn	K-nearest neighbor estimation of Mutual Information Rate
surrogate generation	
surr_ShuffColumn	Create surrogate observation matrices by applying joint random permutation of selected columns
surr_Iaaft	Create surrogate data for a given single process using the IAAFT algorithm
surr_IaaftBivariate	Create surrogate data for a given bivariate process using the IAAFT algorithm

The parameters for the main estimation functions *surr_ISknn* and *surr_MIRknn* are the following:

• *Y* - the analyzed multivariate process, organized as a matrix with rows as samples and columns as processes,
• *V* - the assigned embedding vector,
• *jj* - index of the column for one investigated process,
• *ii* - index of the column for the second investigated process (MIR only),
• *k* - number of neighbors for the KNN estimation,
• *metric* - distance metric for the KNN estimation, and
• *surr* - parameter determining which type of surrogate to be created before estimating the measure.

To enable the creation of the proposed surrogates, the functionality is controlled with the *surr* parameter, where for *surr* = 1 the function applies the shuffling method on defined columns of the observation matrix, for *surr* = 2 applies the IAAFT method, and for *surr* = 0 it does not apply any surrogate method and the measure is estimated from the original data.

In order to demonstrate the functionality of the proposed methods, 2 demo scripts are placed within the root directory of the toolbox, */SID*, along with a single sample data obtained from (Faes et al., 2011) concerning the RR and respiration series.

In both *demo_IS_estimation* and *demo_MIR_estimation*, we demonstrate the steps to evaluate the significance of the measure and the presence of nonlinearities within the RR series and respiration series. The script goes through a basic collection of steps which can be condensed as:

• loading and applying z-score normalization on the data,
• defining the parameters needed for the measure estimations,
• applying the measure estimation function with the *surr* parameter 0,
• applying the measure estimation function with the *surr* parameter 1 and 2, repeated *num_surrogates* times,
• obtaining the percentile values and comparing them to the originally estimated measure.

The potential users can easily adapt to the scripts with their data, parameters, or other changes. The SID toolbox is freely available at the Biosignals and Information Theory Laboratory (BIT Lab) website and https://github.com/helderpinto97/SID_Toolbox along with the real data application and the codes for the full simulation setup.

## 4 Simulations studies

In this section, the surrogate methods presented in Section 2 will be tested in simulations of both univariate and bivariate systems, including linear and nonlinear models. Specifically, for univariate processes, simulations were conducted using an AutoRegressive (AR) model and the Hénon-Hénon map to assess the presence of auto-dependencies and nonlinear dynamics. Subsequently, surrogates designed for detecting coupling and nonlinearities in bivariate systems were applied to a Vector AR model and the Coupled Hénon-Hénon Map. For each simulation model, a total of *M* = 100 surrogates were constructed and a significance level of *α* = 0.05 was considered. For KNN estimation, *k* = 10 neighbors and an embedding dimension of *q* = 2 were applied. These are the values typically employed in the literature for KNN estimation. The choice of *k* involves a trade-off between variance and bias: increasing *k* reduces the variance but increases the bias. A low embedding was used to mitigate the bias on the estimates associated with the *curse of dimensionality* (Bellman, 1966).

### 4.1 Detection of self-dependencies and nonlinear dynamics in univariate processes

#### 4.1.1 AutoRegressive model

The identification of auto dependencies and the presence of nonlinear dynamics is initially tested through the use of IS in conjunction with the surrogate analysis techniques introduced earlier in the AR model (Brockwell and Davis, 2009)

$$X_{n}=2\rho_{x}\cos\left(2\pi f_{x}\right)X_{n-1}-\rho_{x}^{2}X_{n-2}+\varepsilon_{n},$$ (23)

where *f*_*x*_ = 0.3, the pole radius *ρ*_*x*_ was varied from 0 to 0.95 in steps of 0.05, to ensure the stability of the generated models (Barnett and Seth, 2014), and *ɛ*_*n*_ denotes white Gaussian noise with zero mean and unit variance. For each value of *ρ*_*x*_, 100 realizations of length *N* = 1000 were generated.

**FIGURE 3:**
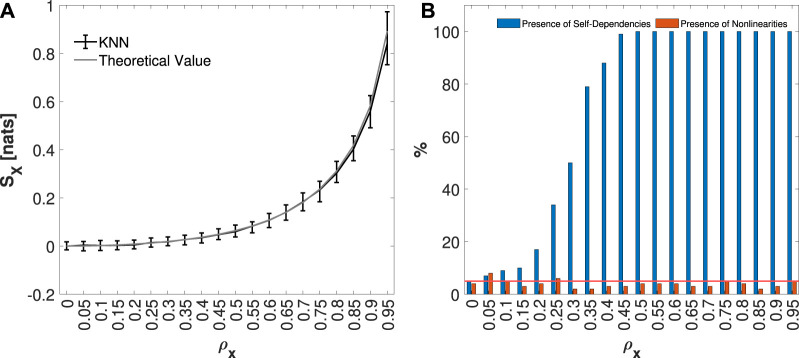
Distribution of IS KNN estimates, median and 5%–95% percentiles, computed on 100 simulations of a univariate AR model, varying the pole radius *ρ*_*x*_ from 0 to 0.95, is shown in panel **(A)**. In panel **(B)**, the plots depict the percentage of realizations, out of 100 realizations for each value of the pole radius *ρ*_*x*_, in which the IS was detected as statistically significant (in blue), and for which nonlinearities (in orange) were found. The red line represents the 5% significance level.

The median and 5%–95% percentile range of the IS values computed for the AR model, for each *ρ*_*x*_ considered, are presented in Figure 3A. Since the AR model is a Gaussian process it is possible to obtain the theoretical value of IS, which is represented, in the same panel, in gray color. In Figure 3B, the blue bars indicate the percentage of the 100 generated realizations in which IS was detected as statistically significant for each *ρ*_*x*_ value, while the orange bars represent the fraction of realizations in which nonlinearities were identified by the IAFFT algorithm. As *ρ*_*x*_ increases, the regularity of the process increases, leading to an increase in IS, as illustrated in Figure 3A. Correspondingly, Figure 3B shows an increase in the significance of the measure, represented by the blue bars. On the other hand, since the AR model is linear, only a small percentage of realizations exhibit nonlinearities, as indicated by the orange bars, showing that the rate of false detection oscillates around the nominal 5% level.

#### 4.1.2 Hénon map

The univariate Hénon Map, as defined by (Hénon, 1976), is described by the following equation

$$X_{n}=1-1.4X_{n-1}^{2}+0.3X_{n-2}.$$ (24)

For this simulation, the process *Y*, defined as

Y=X+ξ, (25)

was used, where *ξ* denotes Gaussian noise with zero mean and variance *σ*. By incrementally increasing the noise variance from 0 to 3 in steps of 0.2, the past dependencies, as well as the nonlinear dynamics of the Hénon Map defined in Equation 24 (considering the classic parameters for chaotic behavior), are masked. This approach enables the assessment of surrogate methods proposed to test the statistical significance of IS and the presence of nonlinear dynamics. For each value of *σ* considered, 100 simulations of length *N* = 1000, using a burn-in period of *N*_*trans*_ = 1000 to avoid transient behavior, were computed using random initial conditions.

**FIGURE 4:**
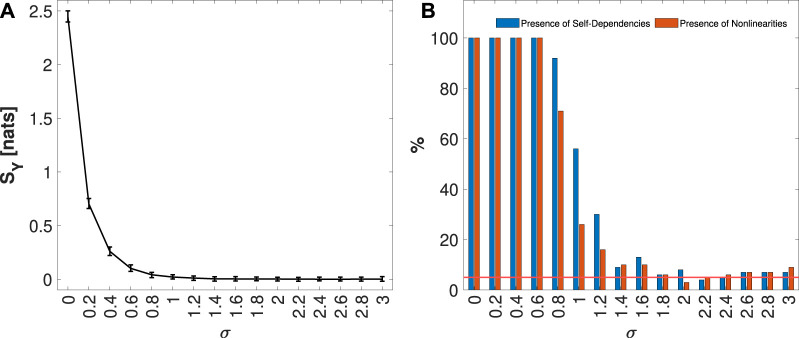
Distribution of IS KNN estimates, median and 5%–95% percentile range, computed on 100 simulations of *Y* process defined in Eq. 25, varying the variance noise *σ* from 0 to 3, is shown in panel **(A)**. In panel **(B)**, the plots depict the percentage of realizations, out of 100 simulation runs for each value of *σ*, in which the IS was detected as statistically significant (in blue), and for which nonlinearities (in orange) were found. The red line represents the 5% significance level.

Figure 4A presents the median and 5%–95% percentile range of the IS values computed for the process *Y* formulated in Eq. 25, for each value of the noise variance *σ* considered. In Figure 4B, the blue bars indicate the percentage of the 100 generated realizations in which IS was detected as statistically significant, while the orange bars represent the fraction for which the IAFFT algorithm detected nonlinearities. The increase in noise variance conceals the dependence between the present and the past of the process, leading to a reduction of the information storage, as illustrated in Figure 4A. Consequently, Figure 4B shows a decrease in the presence of statistically significant self-dependencies. Additionally, the higher noise variance masks nonlinearities, contributing to the observed downward trend in the presence of nonlinear dynamics. In summary, these results, together with those discussed in Section 4.1.1, highlight the effectiveness of the surrogate methods proposed in this work for detecting self-dependencies and the presence of nonlinearities in univariate processes.

### 4.2 Detection of dynamic coupling and nonlinear interactions in bivariate processes

#### 4.2.1 Vector AutoRegressive model

The surrogate procedures for the significance and the presence of nonlinear coupling using the MIR are first tested in a stable bivariate Vector AutoRegressive (VAR) unidirectionally coupled model defined as (Barnett and Seth, 2014; Faes et al., 2015)

$$\begin{array}{cc} & X_{n}=2\rho_{x}\cos\left(2\pi f_{x}\right)X_{n-1}-\rho_{x}^{2}X_{n-2}+\varepsilon_{n},\\ & Y_{n}=2\rho_{y}\cos\left(2\pi f_{y}\right)Y_{n-1}-\rho_{y}^{2}Y_{n-2}+C\cdot X_{n-2}+\xi_{n}, \end{array}$$ (26)

where *ɛ*_*n*_ and *ξ*_*n*_ are independent white Gaussian noises with zero mean and unit variance, and *ρ*_*x*_ = 0.3, *f*_*x*_ = 0.3 and *ρ*_*y*_ = 0.3, *f*_*y*_ = 0.1. In this simulation, *X* and *Y* exhibit second-order autoregressive behavior, characterized by two complex-conjugate poles with a modulus of *ρ*_*x*,*y*_ and phases of ± 2*πf*_*x*,*y*_. This configuration establishes autonomous wide-band oscillations at frequencies of 0.3 and 0.1 Hz for *X* and *Y*, respectively. Directional connections are set from *X* to *Y*, at lag 2, modulated by the parameter *C*. For each value of the coupling parameter *C*, which varies between 0 and 1 in steps of 0.05, 100 realizations of length *N* = 1000 were generated.

**FIGURE 5:**
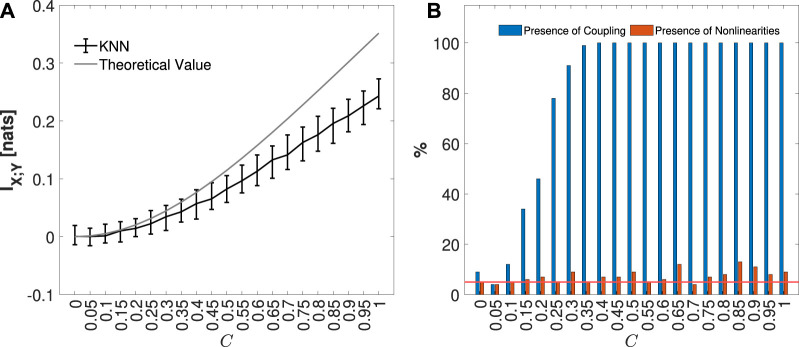
The distribution of MIR KNN estimates, median, and 5%–95% percentile range, computed on 100 simulations of the VAR process, varying the coupling parameter *C* from 0 to 1, is shown in black, along with the respective theoretical values in gray, in panel **(A)**. In panel **(B)**, the plots depict the percentage of realizations, out of 100 simulation runs for each value of *C*, in which the IS MIR detected as statistically significant (in blue), and for which nonlinearities (in orange) were found. The red line represents the 5% significance level.

**FIGURE 6:**
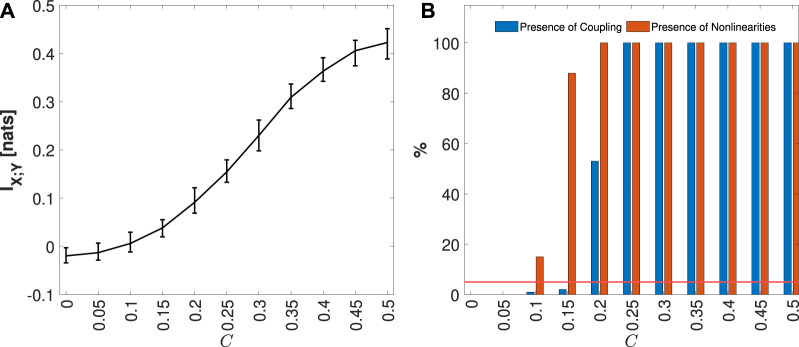
Distribution of MIR KNN estimates, median and 5%–95% percentile range, computed on 100 simulations of Coupled Hénon-Hénon Map, varying the coupling parameter C from 0 to 0.5, is shown in panel **(A)**. In panel **(B)**, the plots depict the percentage of realizations, out of 100 simulation runs for each value of *C*, in which the IS MIR detected as statistically significant (in blue), and for which nonlinearities (in orange) were found. The red line represents the 5% significance level.

The median and 5%–95% percentile range of the MIR values computed for the VAR model, considering each coupling parameter *C*, are presented in Figure 5A. In Figure 5B, the blue bars indicate the percentage of the 100 generated realizations in which MIR was detected as statistically significant, while the orange bars represent the fraction of simulations in which nonlinearities were identified. As expected, the increase in the parameter *C* leads to an increase in I_*X*;*Y*_, as reported in Figure 5A. It is also important to note that for low values of *C*, negative values of MIR are observed. This can be attributed to the bias of the KNN estimator (Kraskov et al., 2004; Mijatovic et al., 2021), which tends to underestimate the MIR, resulting in values near zero becoming negative but not statistically significant, as illustrated in Figure 6B. Consequently, in Figure 5B, the presence of significant coupling increases along with the coupling parameter. On the other hand, due to the linearity of the VAR model, there is a low fraction of realizations, distributed around the nominal 5% error rate, where nonlinearities were detected for each *C* value considered.

#### 4.2.2 Coupled Hénon-Hénon maps

The unidirectionally coupled Hénon-Hénon system is described by the following equations (Sausedo-Solorio and Pisarchik, 2011)

$$\begin{array}{cc} & X_{n}=1.4-X_{n-1}^{2}+0.3X_{n-2},\\ & Y_{n}=1.4-\left(CX_{n-1}Y_{n-1}+\left(1-C\right)Y_{n-1}^{2}\right)+0.3Y_{n-2}. \end{array}$$ (27)

For this simulation, the coupling parameter *C* was varied from 0 to 0.5 in steps of 0.1. The selection of this interval for *C* is primarily based on the system manifestation of chaotic behavior within this range, without the occurrence of synchronization, which is observed at higher values of the coupling parameter (Krakovská et al., 2018). For each *C*, 100 realizations of length *N* = 1000 with a burn-in period of *N*_*trans*_ = 1000 to avoid the transitory behavior were computed using random initial conditions. As the parameter *C* increases, the nonlinear coupling increases, on the other hand, the internal dynamics of *Y* is reduced.

Figure 6A presents the median and 5%–95% percentile range of the MIR values computed for the Coupled Hénon-Hénon Map, considering each coupling parameter *C*. In Figure 6B, the blue bars depict the percentage of the 100 generated realizations where MIR was detected as statistically significant, while the orange bars represent the fraction of simulations in which nonlinearities were identified. As expected, with the increase in the coupling parameter *C*, the *I*_*X*;*Y*_ rises, as observed in Figure 6A. This observation is further highlighted in Figure 6B, illustrating the significance of this measure and the increased detection of nonlinear coupling. As in the case of the VAR model, for low coupling values, some realizations exhibit negative values due to the bias of the KNN estimator (Kraskov et al., 2004; Mijatovic et al., 2021).

### 4.3 Estimators complexity

In this section the execution time of the two main functions surr_ISknn and surr_MIRknn, introduced in Section 3, are analyzed in the AR and VAR model, introduced, respectively, in Sections 4.1.1, 4.2.1. The analysis was carried out on a PC with Windows 11 Home OS and an Intel(R) Core(TM) i7-10750H CPU @ 2.60GHz, with 16 GB of RAM and using MATLAB (R2023b) Update 7.

**FIGURE 7:**
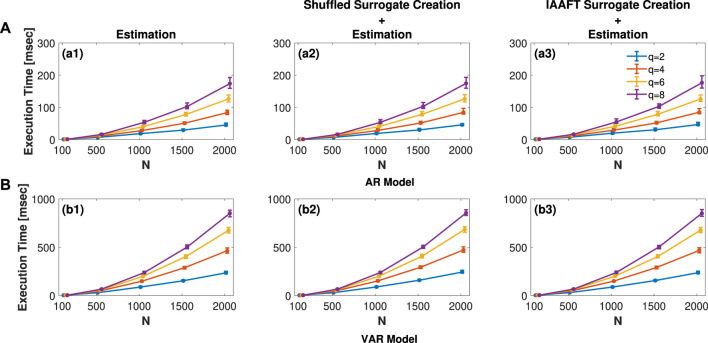
Distribution of execution time in msec of **(A)** surr_ISknn and **(B)** surr_MIRknn performing only the estimation procedure. The plots present median and 5%–95% percentiles, computed as follows: in **(A)**, based on 100 simulations of a univariate AR model (Eq. 23) with *ρ*_*x*_ =0.5 and *f*_*x*_ =0.3; and in **(B)**, across 100 simulations of the VAR model (Eq. 26) with *ρ*_*x*_ =0.3, *f*_*x*_ =0.3, *ρ*_*y*_ =0.3, *f*_*y*_ =0.1, and *C* =0.5. For both univariate and bivariate cases, signal lengths *N* = 100, 500, 1000, 1500, 2000, and embedding dimensions *q* = 2, 4, 6, 8 were considered.

Figure 7 illustrates the distribution of execution times for surr_ISknn in panel **(a)** and surr_MIRknn in panel **(b)**. Each plot displays the median and 5%–95% percentiles, calculated as follows: in **(a)**, based on 100 simulations of a univariate AR model with *ρ*_*x*_ = 0.5 and *f*_*x*_ = 0.3; and in **(b)**, derived from 100 simulations of the VAR model with *ρ*_*x*_ = 0.3, *f*_*x*_ = 0.3, *ρ*_*y*_ = 0.3, *f*_*y*_ = 0.1, and *C* = 0.5. Both cases considered signal lengths *N* = 100, 500, 1000, 1500, 2000, and embedding dimensions *q* = 2, 4, 6, 8.

As expected, the execution time of surr_MIRknn, illustrated in Figure 7A, is higher than that of surr_ISknn, presented in Figure 7B, since the former is employed in bivariate systems where the complexity of calculations is superior. Additionally, for both functions, increasing the signal length results in more time consumed in the range search, leading to an expected increase in execution time, as observed. Similarly, when increasing the embedding dimension *q*, the dimension of the observation matrix becomes greater, consequently requiring more time for estimation.

One important point to stress is that most of the time consumed is for KNN estimation of the information measures. If some of the surrogate procedures proposed herein are used, a small increment in the results presented in Figure 7 will be observed. The magnitude of this increment is expected to be proportional to the number of surrogates utilized for the test. In particular, for the univariate case, under the worst-case scenario with *N* = 2000 and *q* = 8, we observe a median increase in execution time of approximately 1 ms for the shuffled surrogate creation followed by IS estimation, illustrated in Figure 7(a2). Conversely, when using IAAFT surrogates followed by IS estimation, there is an increment in execution time of approximately 3 ms, presented in Figure 7(a3). In the case of the VAR model, with *N* = 2000 and *q* = 8, there is a median increase in time of approximately 4 ms for the creation of shuffled surrogates along with the MIR estimation as presented in Figure 7(b2). Conversely, when utilizing IAAFT surrogates with MIR estimation to assess the presence of nonlinear dynamics, a median increase of approximately 2 ms is observed in Figure 7(b3).

## 5 Analysis of cardiorespiratory interactions

This section reports the application of the surrogate approaches proposed to evaluate the presence of self-dependencies, nonlinear dynamics, and coupling, as presented in Sections 2.2, 2.3, 2.4, 2.5, in a physiological dataset. The aim is to prove the effectiveness of the proposed approaches for assessing the cardiorespiratory interactions during both spontaneous and paced breathing.

### 5.1 Experimental protocol

Cardiorespiratory interactions were explored considering a historical database collected for analyzing short-term physiological variability during paced breathing (Faes et al., 2011). The experiments were conducted at the Cardiology Unit of S. Chiara Hospital in Trento, Italy. All participants provided informed consent, and the experimental protocol received approval from the hospital Ethical Committee. The dataset comprises physiological time series obtained from 16 young and healthy individuals observed in the supine position during four distinct experimental conditions: spontaneous breathing (SB), controlled breathing at 10 breaths/min (C10), at 15 breaths/min (C15), and at 20 breaths/min (C20). The acquired signals were the surface electrocardiographic signal (ECG, lead II) and the respiratory nasal flow (by differential pressure transducer). Signals were collected simultaneously and digitized at 1 kHz sampling rate and 12-bit precision. The sequence of heart periods (RR intervals) was extracted from the ECG signal, representing the time intervals between two consecutive R peaks in the ECG. The respiratory amplitude values (RESP) were extracted from the nasal respiration flow signal sampled at the onset of each heart period. The analysis involved synchronous time series consisting of 300 values for each subject and condition.

### 5.2 Statistical analysis

Significant changes in the IS and MIR across the pairs of experimental conditions were assessed using repeated measures models (Davis, 2002; Crowder and Hand, 2017; Davidian, 2017). Estimated marginal means (EMM) were calculated between each paced breathing condition and the spontaneous (SB vs. C10, SB vs. C15, and SB vs. C20) (Searle and Milliken, 1980). A *Z*-test is then applied to determine the significance of these differences at a significance level of *p* < 0.05, with the Bonferroni correction applied for multiple comparisons (*n* = 3). The model’s residuals were checked for whiteness. MATLAB software (MathWorks, Natick, MA, United States) was used to build the models and compute EMM.

### 5.3 Results of real data analysis

**FIGURE 8:**
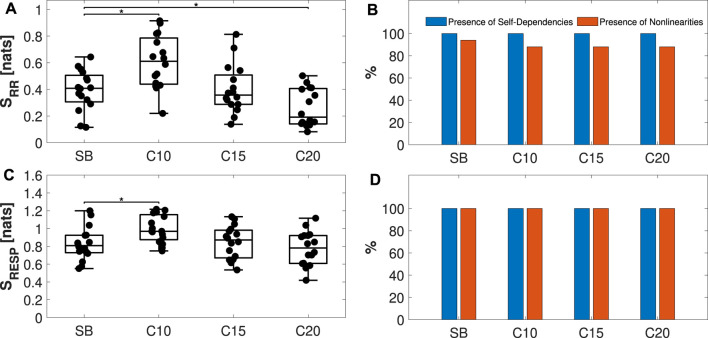
Analysis of the individual dynamics of physiological variability time series. Panels **(A, C)** display boxplot distributions and individual values of the IS computed on RR and RESP time series, respectively, analyzed during spontaneous breathing (SB) and controlled breathing at 10, 15, and 20 breaths/min (C10, C15, C20). On the right, panels **(B, D)** present the percentage of individuals exhibiting self-dependencies (blue) and nonlinearities (orange) when considering RR and RESP processes, respectively. Statistical analysis: *post hoc* test with a Bonferroni correction of the estimated marginal means (EMM) of a repeated measures model:*p < 0.05.

Figures 8A, C present the boxplot distributions with the overlapping individual values of *S*_RR_ and *S*_RESP_, respectively. On the right, Figures 8B, D report the percentage of subjects presenting self-dependencies (in blue) and nonlinearities (in orange) when taking into account the RR and RESP time series, respectively. Results evidence a significant increase in IS from SB to C10, followed by a decrease when going from C10 to C20 with regard to RR, and a statistically significant difference from SB (panel (a)). On the contrary, although the trend is the same, only the increase from SB to C10 is found significant when taking into account RESP time series (panel (c)). The values across all considered phases were statistically significant, indicating the presence of self-dependencies on RR and RESP time series. Additionally, the majority of the tested individuals exhibit nonlinear dynamics, especially for RESP time series, as illustrated in Figure 8D, with a tendency towards decreasing the nonlinearity of the RR series while increasing the frequency of the paced breathing, as presented in Figure 8B.

**FIGURE 9:**
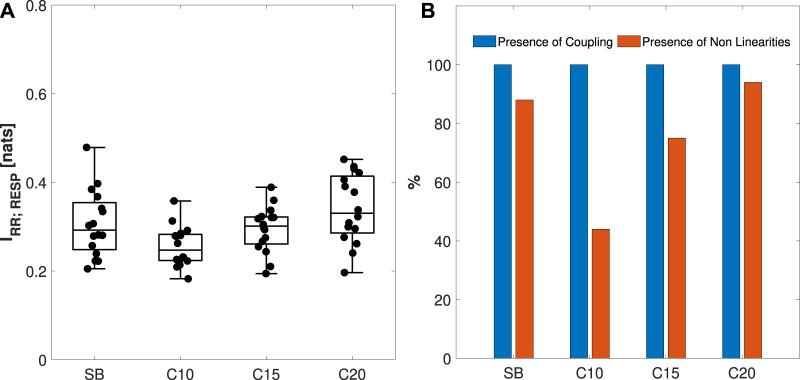
Analysis of the coupled dynamics in the cardiorespiratory system *S* = {RR,RESP}. Panel **(A)** reports the boxplot distributions and individual values of the MIR, computed during spontaneous breathing (SB) and controlled breathing at 10, 15, and 20 breaths/min (C10, C15, C20). Panel **(B)** reports the percentage of individuals for which coupling (blue) and nonlinear dynamics (orange) were found. Statistical analysis using a *post hoc* test with a Bonferroni correction of the estimated marginal means (EMM) of a repeated measures model:*p < 0.05.

In Figure 9A, the distribution and individual values of *I*_*RR*;*RESP*_ for the four experimental conditions are presented. The percentage of subjects exhibiting significant coupling (in blue) and nonlinearities (in orange) is reported in Figure 9B. Figure 9A shows a slight not statistically significant decrease from the SB to the C10 phase followed by the recovery of MIR values in C15 and C20. Nonetheless, all the MIR estimates are statistically significant, as illustrated in Figure 9B. With regard to the presence of nonlinearities (panel (b)), a marked decrease is observed for C10 if compared to SB, followed by a recovery when going to C15 and C20.

### 5.4 Discussion

Firstly, the presence of self-dependencies and nonlinear dynamics was individually analyzed for each of the processes, RR and RESP, using IS. Physiologically, the higher IS detected during C10, as observed in Figures 8A, C, suggests less complex RR and RESP dynamics (Radhakrishna et al., 2000). This observation may be linked to the mechanism of Respiratory Sinus Arrhythmia (RSA), i.e., modulation of heart rate with respiration. The RSA mechanism is more prominent during forced ventilation at low breathing rates (Saul et al., 1989), and its effects, compared to spontaneous breathing, tend to diminish when increasing respiratory rate (Song and Lehrer, 2003). Furthermore, RSA and other cardiovascular control mechanisms, such as cardio-ventilatory coupling and synchronization of respiratory stroke volume (Elstad et al., 2018), not only produce alterations in the complexity of respiratory dynamics but also similar variations in the complexity of cardiac dynamics. Nonetheless, a difference is found with regard to the nonlinearities between the two systems: controlled breathing produces a slight decrease of nonlinearities in cardiac dynamics with breathing rate, which is instead not observed in respiratory dynamics (Fang et al., 2008; Dimitriev et al., 2019).

The analysis of the coupling of the bivariate system S = {*RR*, *RESP*} using MIR highlights that the coupling between RR and RESP is not lost during different paced breathing rates (Mary et al., 2018), being the slight decrease observed in Figure 9 from the SB to the C10 phase not significant, and is followed by a complete recovery in C15 and C20. Such results are similar to what was reported in (Barà et al., 2023). A decrease in nonlinearities was observed in the C10 phase when compared with the other experimental conditions. This suggests that lower breathing rates reduce the nonlinearity of cardiorespiratory coupling (Fang et al., 2008), reflecting the possible effect of entrainment between low-frequency and high-frequency (respiration-induced) oscillations of HRV. This entrainment causes the entire HRV pattern to be centered around a single (broad band) stochastic oscillation, resulting in more linear dynamics. The trend differs when comparing *I*_*RR*;*RESP*_ and *S*_*RR*_ in terms of nonlinearities: RR nonlinearities appear to be mainly associated with the controlled breathing condition and seem less related to the frequency rate, unlike *I*_*RR*;*RESP*_ nonlinearities, which appear instead to depend only on the breath rate.

## 6 Conclusion

This study introduces surrogate approaches to examine specific statistical properties in univariate and bivariate dynamic processes. These approaches explore self-dependencies and nonlinearities in univariate processes, along with coupling and nonlinearities in bivariate systems. The proposed methods were validated through simulations and subsequently applied to investigate cardiorespiratory interactions from physiological time series.

The simulation results revealed that the proposed approaches successfully detect key dynamical characteristics of stochastic systems related to the internal properties of individual systems and the coupling structure between two systems, as well as to the nature (linear/nonlinear) of the underlying dynamics. Moreover, the application to real data has allowed to infer interesting different behaviors with regard to cardiorespiratory interactions during spontaneous and paced breathing. In particular, controlled breathing increases the predictability of both RR and RESP dynamics. A decrease in nonlinearities when increasing the breathing rate is observed in cardiac, but not in respiratory dynamics. Moreover, the different paced breathing rates do not alter the cardiorespiratory coupling, while its nonlinearities are reduced during lower breathing rates.

Future developments will aim to test the surrogate methods discussed herein on various biosignals in the context of network physiology (Ivanov, 2021), particularly focusing on the analysis of brain-heart interactions (Koutlis et al., 2021; Antonacci et al., 2023; Varley et al., 2023). The proposed integrated approach for evaluating dynamic dependencies and nonlinearities in univariate and bivariate time series, implemented with the provided toolbox, will foster the assessment of the statistical temporal structure in coupled processes within Network Physiology and its related fields.

## Data Availability

The data analyzed in this study is subject to the following licenses/restrictions: The physiological raw data can be shared by the corresponding author upon request if data privacy can be guaranteed according to the rules of the European General Data Protection Regulation (EU GDPR). Requests to access these datasets should be directed to LF, luca.faes@unipa.it.
